# Frequent DNA Hypermethylation at the *RASSF1A* and *APC* Gene Loci in Prostate Cancer Patients of Pakistani Origin

**DOI:** 10.1155/2013/627249

**Published:** 2013-01-30

**Authors:** Ahmed Yaqinuddin, Sohail A. Qureshi, Shahid Pervez, Mohammed Umair Bashir, Ressam Nazir, Farhat Abbas

**Affiliations:** ^1^College of Medicine, Alfaisal University, Al Mather Street, Takhassusi Road, Riyadh 11533, Saudi Arabia; ^2^Department of Biology, Syed Babar Ali School of Science and Engineering, Lahore University of Management Sciences, Lahore 54792, Pakistan; ^3^Department of Microbiology and Pathology, The Aga Khan University, Stadium Road, Karachi 74800, Pakistan; ^4^Medical College, The Aga Khan University, Stadium Road, Karachi 74800, Pakistan; ^5^Department of Surgery, The Aga Khan University, Stadium Road, Karachi 74800, Pakistan

## Abstract

DNA methylation has emerged as a potentially robust biomarker for prostate cancer (PCa). Since DNA methylomes appear to be disease as well as population specific, we have assessed the DNA methylation status of *RASSF1A, APC,* and *p16* (potential biomarkers of PCa) in Pakistani population. Primary prostate cancer tissues were obtained from 27 formalin-fixed paraffin-embedded blocks (FFPE) of cancer patients who underwent radical prostatectomy and transurethral resection of prostate (TURP) during 2003–2008. As controls, twenty-four benign prostatic FFPE tissues were obtained from patients who underwent TURP for benign prostatic hyperplasia during 2008. DNA was extracted, and methylation-specific PCR was used to assess the methylation status for *RASSF1A, APC,* and *p16* gene promoters. Our results revealed that the *RASSF1A* promoter was hypermethylated in all the tested cancer samples but was also hypermethylated in 3 out of 24 control tissues. The *APC* promoter was hypermethylated in 15 out of 27 cancer samples and in none of the control samples. Strikingly, none of the samples showed methylation at the *p16* promoter. Our findings suggest that *RASSF1A* and *APC* gene promoters are frequently hypermethylated in the Pakistani population and therefore have the potential to develop into universally dependable biomarkers for detecting PCa.

## 1. Introduction

A well-characterized epigenetic mechanism is DNA methylation. DNA methylation typically occurs at CpG islands that are located in the promoter regions of about 50% of human genes. In general, hypermethylation of gene regulating regions (promoters) turns off gene expression, whereas hypomethylation has the opposite effect. Unprogrammed changes in the DNA methylome can culminate in the establishment of a disease state by activating or repressing genes related to cell cycle, growth, and apoptosis [1–3]. Aberrant DNA methylation profile (either hyper or hypo) has been linked to variety of malignancies and emerged as a potentially useful biomarker for monitoring neoplasia [4–6].

Current methods of detecting PCa including PSA and transrectal biopsy fall far short to be ideal methods for diagnosing clinically significant PCa [7, 8]. Epigenomic alterations appear to contribute significantly to PCa onset. A number of gene promoters including *GSTP1, APC, RASSSF1A, COX2, MDR1, ER α, hMLH1,* and *p14/INK* have also been found to be frequently hypermethylated in PCa [9–12]. Accumulating data on the methylation status of various genes indicates that biomarkers based on specific methylomes may serve to differentiate between cancerous and non-cancerous prostate tissues [9, 12–14]. 

One caveat that is beginning to emerge as more information is becoming available on alteration in DNA methylomes and PCa is that these changes are not only disease specific but also population specific. An example of this phenomenon is illustrated by the difference in the relationship between methylation status of *p16* gene in Japanese population versus Caucasian population (hypermethylation in Japanese and hypomethylation in Caucasians [10, 12]). 

Further studies on different geographical populations are warranted to provide rational basis for development of both universal as well as population specific biomarkers. 

Herein, we assessed the DNA methylation status of *APC and RASSF1A* (hypermethylated in all populations) and *p16* (variable methylation status) in Pakistani PCa patients.

## 2. Materials and Methods

### 2.1. Tissues

Prostate cancer tissues were obtained from 18 paraffin-embedded blocks of cancer patients who underwent radical prostatectomy and 9 patients who underwent transurethral resection of prostate (TURP) during 2003–2008; blocks with >70% cancerous tissue were selected after histological examination of slides. Gleason score, tumor stage and serum PSA values were collected for each subject at the time of surgery. Twenty-four benign prostate tissues were obtained from paraffin-embedded blocks of patients who underwent transurethral resection of prostate (TURP) for benign enlargement of prostate gland during 2008 and used as controls. All samples were collected following the protocol approved by Ethical Review Committee of Aga Khan University Hospital, Karachi.

### 2.2. DNA Isolation and Bisulphite Conversion

DNA was isolated from paraffin-embedded blocks with DNA extraction kit (Qiagen) according to the manufacturer's protocol. About 2 *μ*g of genomic DNA was subsequently subjected to sodium bisulphite modification using Methyl-Easy DNA bisulphite modification kit (Human Genetic Signatures, Australia) using the manufacturer's protocol. 

### 2.3. Methylation-Specific PCR (MSP)

2 *μ*L of the bisulphite-converted DNA was used as template for MSP. Primer sequences for amplifying *RASSF1A, APC,* and *p16* were described previously. [15] All PCRs were carried on Mastercycler (Eppendorf) using the following cycling conditions: 95°C for 10 minutes; 40 cycles of 94°C for 30 seconds, 55°C for 30 seconds; 72°C for 30 seconds, with a final extension at 72°C for 7 minutes. Bisulphite-converted SssI methylase-treated WBC DNA was employed as a positive control, while untreated-WBC (bisulphite-converted) DNA served as negative control.

### 2.4. Statistical Analysis

Using CpG island methylation data for 27 primary prostate tumors and 24 controls, the optimal sensitivity and specificity of each DNA methylation marker was determined independently and in combination. A measure of differentiation of PCa was coded such that all tumors with cumulative Gleason score 7 or less were taken as differentiated, while tumors with a >7 score were considered undifferentiated aggressive cancers. Mann-Whitney *U* test was applied to look at the statistical significance of frequency of hypermethylation of candidate genes. A Fisher-exact test was run to determine the association between the methylation status of the candidate genes and stage of cancer. These tests were run using SPSS 16.0 (statistical software package).

## 3. Results

The demographics of prostate cancer patients who contributed to this study are shown in Table 1. MSP was performed to evaluate methylation status of the three genes. Our results show that *RASSF1A *was hypermethylated in all 27 prostate cancer tissues. Unexpectedly, the *RASSF1A* promoter was also hypermethylated in 3 of the 24 benign prostatic (i.e., control) tissues samples (Tables 2 and 3). Hypermethylation at the *APC* promoter was observed in 58% (i.e., 15 out of 27) of the cancer cases (Tables 2 and 4) but in none of the benign prostatic tissues. The promoter of *p16 *genes showed no hypermethylation in cancerous or control samples.

Since a collection of benign and primary prostate cancer tissues were studied, sensitivity and specificity of each of the gene markers were calculated to assess whether it is capable of distinguishing primary prostate cancer from benign prostatic hyperplasia. The sensitivity and specificity of *RASSF1A* and *APC* are given as Table 5. The sensitivity and specificity increase to 100% if we use *RASSF1A* and *APC* in combination.

 A Fisher's exact test was performed to assess the association of stage of tumor with the methylation status of the three genes. This analysis showed that *APC* hypermethylation was associated with early stage tumors with a *P* value of 0.049 (Table 4). 

## 4. Discussion

This is the first report to our knowledge that showed hypermethylation of *RASSF1A* and *APC* gene promoters in the prostate cancer samples derived from a Pakistani population. Further, the use of both *RASSF1A *and *APC* in combination showed greater sensitivity and specificity to detect PCa in our population. We described for the first time that hypermethylation of *APC *gene locus occurs early in the development of PCa in our population. Strikingly, our results demonstrated no methylation at the *p16* locus contrary to studies conducted by the Japanese group [10].

In the present study, frequency of hypermethylation at *RASSF1A *and *APC* loci in PCa patients was ~100% and ~55%, respectively. In general, these observations are consistent with previous studies. However, previous studies in different geographical populations showed variable frequency distribution of methylation at these loci [9, 11, 12]. For example, the variability in frequency of hypermethylation of *RASSF1A* gene promoters was found to be between 58% and 95% in different populations [3, 9, 16, 17], and for *APC,* this variability was even greater between 27% and 95% [3, 17–19]. Such differences in DNA methylation frequencies are likely due to one or more of the following: disease heterogeneity, grade of cancer sample and ethnicity of the PCa patients who were involved in these studies [3, 20]. Collectively, all these studies performed in different geographical populations including ours in Pakistani population highlight the potential of using hypermethylation of *RASSF1A* and *APC *as universal biomarkers for PCa detection.

Employing two or more genes as biomarkers has been found to significantly increase the sensitivity as well as specificity of the detection test [9, 16]. Jerónimo et al. reported that the combined use of *GSTP1* and *APC* DNA methylation levels increased detection rate of prostate adenocarcinoma significantly as compared to using *GSTP1 *alone [9]. Likewise, our results also demonstrated that the combined use of *RASSF1A* and *APC *can yield into a more sensitive and specific tool to detect PCa in Pakistani population.

The Japanese and Chinese groups have previously reported hypermethylation of *p16 *locus in their sets of PCas [10, 21]. By contrast, studies looking at the methylation status of this gene locus in the Caucasian population showed hypomethylation. Strikingly, our results showed no methylation at this locus in any of the PCa samples. Therefore, methylomes in different geographical populations can be variable, and not every gene locus which is frequently hypermethylated in one geographical population can be used as a universal biomarker for detection of PCa. 

Hypermethylation at* APC* locus has been shown to be associated with cancer progression and the Gleason score [22]. Our results showed that *APC* was frequently methylated at the early stage of PCa. Thus, our study showed that *APC* hypermethylation can be used as a biomarker to detect early stage PCas. 

## 5. Conclusion

Although small in size, this study is the first of its kind on a Pakistani population. Our findings that *RASSF1A *and *APC *genes are frequently methylated in Pakistani PCa patients, demonstrating that both loci could be considered as universal DNA methylation biomarkers for PCa detection. However, further verification of our findings will require a larger sample size with an evenly distributed proportion of early and advanced stage prostate cancer tissues to develop methylation markers which can predict the outcomes of PCa. 

## Figures and Tables

**Table 1:** Clinical characteristics of prostate cancer and benign prostatic hyperplasia patients.

Clinical variable	Prostate cancer patients	BPH patients
Age (y)	67 ± 7	66 ± 4
Mean	67	66
Median	70	66.5
Range	55–78	53–78
Preoperative PSA (ng/mL)		
Median	13	2.98
Range	1.2–487	0.65–65
TNM stage		
Stage T1a	0 (0%)	
Stage T1b	4 (15%)	
Stage T1c	3 (11%)	
Stage T2a	7 (26%)	
Stage T2b	3 (11%)	
Stage T2c	3 (11%)	
Stage T3a	7 (30%)	
Gleason score		
5	2 (7%)	
6	9 (33%)	
7	6 (22%)	
8	4 (16%)	
9	6 (22%)	
Lymph node involvement	2 (7%)	
Seminal vesicle involvement	6 (22%)	
Distant metastasis	4 (15%)	
Total	27	24

**Table 2:** Frequency of hypermethylation of gene loci.

Gene	BPH	PCa	*P* value*
*RASSF1A *	3 (12.5%)	27 (100%)	<0.001
*APC *	0	15 (58%)	<0.001
*p16 *	0	0	

*Mann-Whitney *U* test.

**Table 3:** Association of *RASSF1A* methylation with prostate cancer.

	Methylated	Unmethylated	*P* value*
BPH	3	21	
Pca	27	0	*P* < 0.001

*Fisher's exact test applied.

**Table 4:** Association of *APC* methylation with prostate cancer and with stage of tumor.

	Methylated	Unmethylated	*P* value*
BPH	0	24	
Pca	15	12	*P* < 0.001

Stages T1 and T2	13	7	
Stages T3 and T4	2	5	*P* < 0.049

*Fisher's exact test applied.

**Table 5:** Sensitivity and specificity of gene loci to detect prostate cancer.

Genes	Sensitivity (%)	Specificity (%)
*RASSF1A *	100	87.5
*APC *	55.6	100
